# Bis[4-(dimethyl­amino)pyridinium] tetra­bromidobis(3,4-dichloro­phen­yl)stannate(IV)–1-bromo-3,4-dichloro­benzene (1/1)

**DOI:** 10.1107/S1600536809017590

**Published:** 2009-05-20

**Authors:** Yau Chin Koon, Kong Mun Lo, Seik Weng Ng

**Affiliations:** a Department of Chemistry, University of Malaya, 50603 Kuala Lumpur, Malaysia

## Abstract

The Sn atom in the title substituted pyridinium stannate bromo-3,4-dichloro­benzene solvate, (C_7_H_11_N_2_)_2_[SnBr_4_(C_6_H_3_Cl_2_)_2_]·C_6_H_3_BrCl_2_, lies on a twofold axis within an octa­hedral C_2_Br_4_ donor set. Each cation forms an N—H⋯Br hydrogen bond to one of the Br atoms of the anion. The solvent mol­ecule is disordered about the twofold rotation axis with equal occupancy. The crystal under investigation was non-merohedrally twinned, with a twin component ratio of 0.76:0.24.

## Related literature

For bis­(4-dimethyl­amino­pyridinium) tetra­halido­diorgano­stan­nates, see: Lo & Ng (2008*a* ▶,*b* ▶); Yap *et al.* (2008 ▶). For deconvolution of the diffraction data, see: Spek (2009 ▶).
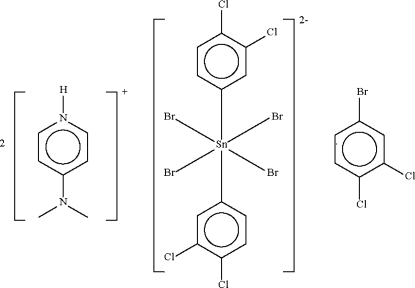


## Experimental

### 

#### Crystal data

(C_7_H_11_N_2_)_2_[SnBr_4_(C_6_H_3_Cl_2_)_2_]·C_6_H_3_BrCl_2_
*M* *_r_* = 1202.55
Monoclinic, 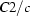

*a* = 19.2308 (2) Å
*b* = 13.8983 (2) Å
*c* = 15.4961 (2) Å
β = 107.491 (1)°
*V* = 3950.23 (9) Å^3^
*Z* = 4
Mo *K*α radiation
μ = 6.14 mm^−1^
*T* = 100 K
0.25 × 0.20 × 0.15 mm

#### Data collection

Bruker SMART APEX diffractometer
Absorption correction: multi-scan (*SADABS*; Sheldrick, 1996 ▶) *T*_min_ = 0.309, *T*_max_ = 0.459 (expected range = 0.268–0.398)
17636 measured reflections
4495 independent reflections
4061 reflections with *I* > 2σ(*I*)
*R*_int_ = 0.030

#### Refinement

*R*[*F* ^2^ > 2σ(*F* ^2^)] = 0.050
*wR*(*F* ^2^) = 0.243
*S* = 1.47
4495 reflections
225 parameters
39 restraints
H-atom parameters constrained
Δρ_max_ = 2.01 e Å^−3^
Δρ_min_ = −1.80 e Å^−3^

### 

Data collection: *APEX2* (Bruker, 2007 ▶); cell refinement: *SAINT* (Bruker, 2007 ▶); data reduction: *SAINT*; program(s) used to solve structure: *SHELXS97* (Sheldrick, 2008 ▶); program(s) used to refine structure: *SHELXL97* (Sheldrick, 2008 ▶); molecular graphics: *X-SEED* (Barbour, 2001 ▶); software used to prepare material for publication: *publCIF* (Westrip, 2009 ▶).

## Supplementary Material

Crystal structure: contains datablocks global, I. DOI: 10.1107/S1600536809017590/tk2446sup1.cif

Structure factors: contains datablocks I. DOI: 10.1107/S1600536809017590/tk2446Isup2.hkl

Additional supplementary materials:  crystallographic information; 3D view; checkCIF report

## Figures and Tables

**Table 1:** Hydrogen-bond geometry (Å, °)

*D*—H⋯*A*	*D*—H	H⋯*A*	*D*⋯*A*	*D*—H⋯*A*
N1—H1⋯Br1	0.88	2.58	3.315 (3)	142

**Fig. 1.**
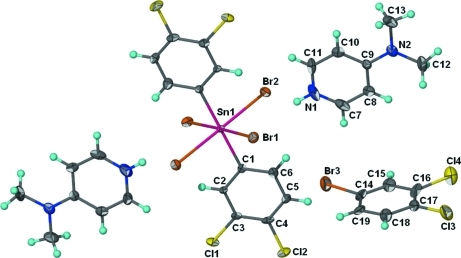
70% Probability thermal ellipsoid plot of the ion-pair (C7H11N2)2 [SnBr4(C6H3Cl2)2].C6H3BrCl2. Unlabelled atoms are related by a 2-fold axis. Hydrogen atoms are drawn as spheres of arbitrary radius.

## supplementary crystallographic information

### Experimental

Tetrakis(3,4-dichlorophenyl)tin (0.70 g, 1 mol) and 4-dimethylaminopyridine hydrobromide perbromide (0.73 g, 2 mmol) were heated in ethanol/chloroform (1:1 *v*/*v*, 100 ml) for 3 h. Crystals separated from the cool solution after a day.

The presence of bromo-3,4-dichlorobenzene in the crystal structure probably arose from contamination of the tetrakis(3,4-dichlorophenyl)tin reactant, which itself was synthesized in a Grignard reaction with bromo-3,4-dichlorobenzene as the starting halogen-bearing compound.

### Refinement

The structure initially refined to 7.7%. PLATON (Spek, 2009) gave the twin law as (1 0 0.746, 0 - 1 0, 0 0 - 1); a new *hkl* file was generated by using the detwinning tool in the program.

The aromatic and pyridyl rings were refined as rigid hexagons of 1.39 Å sides. For the lattice solvent molecule, which is situated about a 2-fold axis, the C–Cl distance was restrained to 1.74±0.01 Å and the C–Br distance to 1.90±0.01 Å. The molecule was allowed to refine off the 2-fold rotation axis. The anisotropic displacement factors of the carbon atoms were restrained to be nearly isotropic.

*SHELXL*-97 suggested an unusually large values for a and b in the weighting scheme, and so the suggested scheme was not used. Instead, an arbitrary value of a = 0.15 was used which gave a statisfactory Goodness-of-Fit of about 1.5.

Hydrogen atoms were placed in calculated positions (C—H 0.95, N–H 0.88 Å) and were included in the refinement in the riding model approximation, with *U*(H) set to 1.2*U*(*C*,*N*). The torsion angles of the methyl groups were refined.

The final difference Fourier map had a large peak at 1.3 Å from H7 and a deep hole at 1.5 Å from H15.

### Crystal data

**Table:**

(C_7_H_11_N_2_)_2_[SnBr_4_(C_6_H_3_Cl_2_)_2_]·C_6_H_3_BrCl_2_	*F*(000) = 2312
*M**_r_* = 1202.55	*D*_x_ = 2.022 Mg m^−^^3^
Monoclinic, *C*2/*c*	Mo *K*α radiation, λ = 0.71073 Å
Hall symbol: -C 2yc	Cell parameters from 9914 reflections
*a* = 19.2308 (2) Å	θ = 2.2–28.4°
*b* = 13.8983 (2) Å	µ = 6.14 mm^−^^1^
*c* = 15.4961 (2) Å	*T* = 100 K
β = 107.491 (1)°	Block, colorless
*V* = 3950.23 (9) Å^3^	0.25 × 0.20 × 0.15 mm
*Z* = 4	

### Data collection

**Table:**

Bruker SMART APEX diffractometer	4495 independent reflections
Radiation source: fine-focus sealed tube	4061 reflections with *I* > 2σ(*I*)
graphite	*R*_int_ = 0.030
ω scans	θ_max_ = 27.5°, θ_min_ = 1.8°
Absorption correction: multi-scan (*SADABS*; Sheldrick, 1996)	*h* = −24→24
*T*_min_ = 0.309, *T*_max_ = 0.459	*k* = −18→18
17636 measured reflections	*l* = −20→20

### Refinement

**Table:**

Refinement on *F*^2^	Primary atom site location: structure-invariant direct methods
Least-squares matrix: full	Secondary atom site location: difference Fourier map
*R*[*F*^2^ > 2σ(*F*^2^)] = 0.050	Hydrogen site location: inferred from neighbouring sites
*wR*(*F*^2^) = 0.243	H-atom parameters constrained
*S* = 1.47	*w* = 1/[σ^2^(*F*_o_^2^) + (0.15*P*)^2^] where *P* = (*F*_o_^2^ + 2*F*_c_^2^)/3
4495 reflections	(Δ/σ)_max_ = 0.001
225 parameters	Δρ_max_ = 2.01 e Å^−^^3^
39 restraints	Δρ_min_ = −1.80 e Å^−^^3^

### Fractional atomic coordinates and isotropic or equivalent isotropic displacement parameters (Å^2^)

**Table:**

	*x*	*y*	*z*	*U*_iso_*/*U*_eq_	Occ. (<1)
Sn1	0.5000	0.61725 (3)	0.7500	0.0092 (2)	
Br2	0.44267 (3)	0.48127 (5)	0.62311 (4)	0.0174 (2)	
Br1	0.44630 (3)	0.75638 (5)	0.62507 (4)	0.0186 (2)	
Cl1	0.81264 (8)	0.62573 (10)	0.85386 (12)	0.0179 (4)	
Cl2	0.81408 (10)	0.62116 (11)	0.65044 (13)	0.0243 (4)	
N2	0.2115 (3)	0.6236 (4)	0.1836 (4)	0.0196 (12)	
C1	0.60066 (16)	0.6174 (2)	0.7153 (3)	0.0102 (11)	
C2	0.6660 (2)	0.6216 (2)	0.7850 (2)	0.0102 (11)	
H2	0.6657	0.6238	0.8461	0.012*	
C3	0.73187 (16)	0.6224 (2)	0.7651 (2)	0.0138 (12)	
C4	0.73233 (17)	0.6191 (3)	0.6756 (3)	0.0155 (13)	
C5	0.6670 (2)	0.6150 (3)	0.6060 (2)	0.0151 (13)	
H5	0.6673	0.6128	0.5449	0.018*	
C6	0.60113 (17)	0.6142 (2)	0.6259 (2)	0.0175 (13)	
H6	0.5564	0.6113	0.5783	0.021*	
N1	0.3539 (2)	0.6345 (3)	0.4428 (2)	0.0293 (14)	
H1	0.3842	0.6371	0.4981	0.035*	
C7	0.38080 (17)	0.6245 (3)	0.3695 (3)	0.0244 (16)	
H7	0.4319	0.6205	0.3790	0.029*	
C8	0.3330 (2)	0.6204 (3)	0.2822 (3)	0.0185 (14)	
H8	0.3514	0.6135	0.2320	0.022*	
C9	0.2582 (2)	0.6262 (3)	0.2682 (2)	0.0144 (12)	
C10	0.23132 (17)	0.6362 (3)	0.3415 (3)	0.0185 (13)	
H10	0.1802	0.6401	0.3320	0.022*	
C11	0.2791 (2)	0.6403 (3)	0.4288 (2)	0.0239 (14)	
H11	0.2608	0.6471	0.4790	0.029*	
C12	0.2386 (5)	0.6177 (5)	0.1057 (5)	0.0273 (17)	
H12A	0.2695	0.6737	0.1050	0.041*	
H12B	0.1974	0.6168	0.0501	0.041*	
H12C	0.2672	0.5587	0.1095	0.041*	
C13	0.1323 (4)	0.6238 (5)	0.1685 (6)	0.0268 (17)	
H13A	0.1196	0.5727	0.2047	0.040*	
H13B	0.1071	0.6126	0.1043	0.040*	
H13C	0.1175	0.6862	0.1866	0.040*	
Br3	0.5258 (5)	0.7963 (5)	0.4343 (4)	0.0355 (11)	0.50
Cl3	0.4624 (2)	0.9970 (3)	0.0505 (2)	0.0351 (9)	0.50
Cl4	0.4620 (14)	0.7751 (14)	0.0633 (11)	0.038 (3)	0.50
C14	0.5139 (16)	0.8551 (8)	0.3234 (8)	0.027 (4)	0.50
C15	0.501 (2)	0.7983 (4)	0.2464 (11)	0.027 (3)	0.50
H15	0.5033	0.7302	0.2517	0.032*	0.50
C16	0.4852 (16)	0.8411 (7)	0.1616 (9)	0.025 (4)	0.50
C17	0.4820 (8)	0.9408 (8)	0.1538 (4)	0.023 (4)	0.50
C18	0.4948 (9)	0.9976 (4)	0.2309 (6)	0.023 (4)	0.50
H18	0.4926	1.0657	0.2255	0.028*	0.50
C19	0.5107 (8)	0.9548 (8)	0.3156 (4)	0.021 (3)	0.50
H19	0.5194	0.9936	0.3683	0.025*	0.50

### Atomic displacement parameters (Å^2^)

**Table:**

	*U*^11^	*U*^22^	*U*^33^	*U*^12^	*U*^13^	*U*^23^
Sn1	0.0068 (4)	0.0113 (4)	0.0099 (3)	0.000	0.0031 (2)	0.000
Br2	0.0141 (4)	0.0186 (4)	0.0195 (4)	−0.0016 (2)	0.0048 (3)	−0.0054 (2)
Br1	0.0175 (4)	0.0215 (4)	0.0169 (4)	0.0013 (2)	0.0050 (3)	0.0030 (2)
Cl1	0.0082 (7)	0.0195 (8)	0.0236 (8)	0.0003 (5)	0.0010 (6)	−0.0001 (5)
Cl2	0.0133 (8)	0.0351 (10)	0.0291 (9)	−0.0020 (5)	0.0132 (7)	−0.0020 (6)
N2	0.019 (3)	0.019 (3)	0.018 (3)	0.0001 (18)	0.001 (2)	0.0001 (19)
C1	0.011 (3)	0.009 (3)	0.011 (3)	0.0016 (17)	0.003 (2)	0.0001 (17)
C2	0.007 (3)	0.014 (3)	0.010 (3)	0.0001 (17)	0.003 (2)	−0.0007 (18)
C3	0.008 (3)	0.014 (3)	0.019 (3)	−0.0021 (18)	0.004 (2)	−0.001 (2)
C4	0.008 (3)	0.015 (3)	0.026 (4)	−0.0011 (18)	0.008 (3)	0.000 (2)
C5	0.016 (3)	0.017 (3)	0.014 (3)	−0.003 (2)	0.007 (2)	−0.002 (2)
C6	0.018 (3)	0.019 (3)	0.016 (3)	−0.002 (2)	0.006 (3)	−0.002 (2)
N1	0.027 (4)	0.033 (3)	0.018 (3)	0.008 (2)	−0.007 (3)	−0.007 (2)
C7	0.016 (3)	0.018 (3)	0.033 (4)	0.003 (2)	−0.002 (3)	−0.006 (3)
C8	0.016 (3)	0.016 (3)	0.023 (3)	0.001 (2)	0.007 (3)	−0.002 (2)
C9	0.017 (3)	0.012 (3)	0.014 (3)	−0.002 (2)	0.003 (2)	−0.0018 (19)
C10	0.015 (3)	0.013 (3)	0.027 (3)	0.000 (2)	0.006 (3)	0.001 (2)
C11	0.033 (4)	0.026 (3)	0.012 (3)	0.008 (3)	0.007 (3)	−0.002 (3)
C12	0.036 (5)	0.031 (4)	0.011 (3)	0.001 (3)	0.001 (3)	0.001 (2)
C13	0.013 (4)	0.032 (4)	0.030 (4)	0.001 (2)	−0.002 (3)	−0.004 (3)
Br3	0.050 (3)	0.036 (3)	0.0186 (11)	−0.0140 (19)	0.0077 (13)	−0.0007 (11)
Cl3	0.049 (2)	0.0289 (19)	0.0208 (16)	−0.0123 (16)	−0.0002 (15)	0.0031 (14)
Cl4	0.047 (7)	0.039 (8)	0.026 (3)	0.008 (4)	0.009 (3)	−0.003 (3)
C14	0.030 (8)	0.036 (7)	0.017 (6)	−0.012 (7)	0.012 (6)	0.003 (6)
C15	0.028 (5)	0.024 (5)	0.032 (5)	0.003 (9)	0.014 (4)	−0.008 (9)
C16	0.018 (7)	0.039 (8)	0.027 (6)	0.014 (6)	0.018 (6)	0.001 (6)
C17	0.024 (6)	0.028 (7)	0.020 (7)	−0.007 (5)	0.012 (5)	−0.009 (6)
C18	0.026 (6)	0.025 (5)	0.019 (9)	0.003 (5)	0.007 (7)	0.000 (4)
C19	0.022 (6)	0.022 (6)	0.023 (7)	−0.007 (5)	0.015 (6)	0.000 (6)

### Geometric parameters (Å, °)

**Table:**

Sn1—C1^i^	2.159 (3)	C8—C9	1.3900
Sn1—C1	2.159 (3)	C8—H8	0.9500
Sn1—Br2	2.7111 (7)	C9—C10	1.3900
Sn1—Br2^i^	2.7111 (7)	C10—C11	1.3900
Sn1—Br1^i^	2.7114 (7)	C10—H10	0.9500
Sn1—Br1	2.7114 (7)	C11—H11	0.9500
Cl1—C3	1.739 (3)	C12—H12A	0.9800
Cl2—C4	1.730 (3)	C12—H12B	0.9800
N2—C9	1.349 (7)	C12—H12C	0.9800
N2—C12	1.454 (10)	C13—H13A	0.9800
N2—C13	1.468 (10)	C13—H13B	0.9800
C1—C2	1.3900	C13—H13C	0.9800
C1—C6	1.3900	Br3—C14	1.855 (6)
C2—C3	1.3900	Cl3—C17	1.719 (7)
C2—H2	0.9500	Cl4—C16	1.718 (9)
C3—C4	1.3900	C14—C15	1.3900
C4—C5	1.3900	C14—C19	1.3900
C5—C6	1.3900	C15—C16	1.3900
C5—H5	0.9500	C15—H15	0.9500
C6—H6	0.9500	C16—C17	1.3900
N1—C7	1.3900	C17—C18	1.3900
N1—C11	1.3900	C18—C19	1.3900
N1—H1	0.8800	C18—H18	0.9500
C7—C8	1.3900	C19—H19	0.9500
C7—H7	0.9500		
			
C1^i^—Sn1—C1	179.87 (18)	C7—C8—C9	120.0
C1^i^—Sn1—Br2	88.95 (10)	C7—C8—H8	120.0
C1—Sn1—Br2	91.15 (10)	C9—C8—H8	120.0
C1^i^—Sn1—Br2^i^	91.15 (10)	N2—C9—C8	120.4 (4)
C1—Sn1—Br2^i^	88.95 (10)	N2—C9—C10	119.6 (4)
Br2—Sn1—Br2^i^	91.62 (3)	C8—C9—C10	120.0
C1^i^—Sn1—Br1^i^	89.95 (10)	C11—C10—C9	120.0
C1—Sn1—Br1^i^	89.95 (10)	C11—C10—H10	120.0
Br2—Sn1—Br1^i^	178.301 (19)	C9—C10—H10	120.0
Br2^i^—Sn1—Br1^i^	89.70 (2)	C10—C11—N1	120.0
C1^i^—Sn1—Br1	89.95 (10)	C10—C11—H11	120.0
C1—Sn1—Br1	89.95 (10)	N1—C11—H11	120.0
Br2—Sn1—Br1	89.70 (2)	N2—C12—H12A	109.5
Br2^i^—Sn1—Br1	178.301 (19)	N2—C12—H12B	109.5
Br1^i^—Sn1—Br1	89.01 (3)	H12A—C12—H12B	109.5
C9—N2—C12	120.5 (6)	N2—C12—H12C	109.5
C9—N2—C13	120.7 (6)	H12A—C12—H12C	109.5
C12—N2—C13	118.8 (6)	H12B—C12—H12C	109.5
C2—C1—C6	120.0	N2—C13—H13A	109.5
C2—C1—Sn1	118.5 (2)	N2—C13—H13B	109.5
C6—C1—Sn1	121.5 (2)	H13A—C13—H13B	109.5
C3—C2—C1	120.0	N2—C13—H13C	109.5
C3—C2—H2	120.0	H13A—C13—H13C	109.5
C1—C2—H2	120.0	H13B—C13—H13C	109.5
C2—C3—C4	120.0	C15—C14—C19	120.0
C2—C3—Cl1	118.8 (2)	C15—C14—Br3	119.1 (9)
C4—C3—Cl1	121.2 (2)	C19—C14—Br3	120.6 (9)
C5—C4—C3	120.0	C16—C15—C14	120.0
C5—C4—Cl2	119.8 (2)	C16—C15—H15	120.0
C3—C4—Cl2	120.2 (2)	C14—C15—H15	120.0
C4—C5—C6	120.0	C15—C16—C17	120.0
C4—C5—H5	120.0	C15—C16—Cl4	122.3 (11)
C6—C5—H5	120.0	C17—C16—Cl4	117.6 (11)
C5—C6—C1	120.0	C18—C17—C16	120.0
C5—C6—H6	120.0	C18—C17—Cl3	118.3 (8)
C1—C6—H6	120.0	C16—C17—Cl3	121.7 (8)
C7—N1—C11	120.0	C17—C18—C19	120.0
C7—N1—H1	120.0	C17—C18—H18	120.0
C11—N1—H1	120.0	C19—C18—H18	120.0
C8—C7—N1	120.0	C18—C19—C14	120.0
C8—C7—H7	120.0	C18—C19—H19	120.0
N1—C7—H7	120.0	C14—C19—H19	120.0
			
Br2—Sn1—C1—C2	−138.45 (15)	C12—N2—C9—C8	−1.9 (6)
Br2^i^—Sn1—C1—C2	−46.86 (16)	C13—N2—C9—C8	176.2 (4)
Br1^i^—Sn1—C1—C2	42.84 (16)	C12—N2—C9—C10	177.2 (4)
Br1—Sn1—C1—C2	131.85 (16)	C13—N2—C9—C10	−4.7 (6)
Br2—Sn1—C1—C6	41.96 (17)	C7—C8—C9—N2	179.0 (4)
Br2^i^—Sn1—C1—C6	133.56 (17)	C7—C8—C9—C10	0.0
Br1^i^—Sn1—C1—C6	−136.74 (17)	N2—C9—C10—C11	−179.0 (4)
Br1—Sn1—C1—C6	−47.73 (17)	C8—C9—C10—C11	0.0
C6—C1—C2—C3	0.0	C9—C10—C11—N1	0.0
Sn1—C1—C2—C3	−179.6 (2)	C7—N1—C11—C10	0.0
C1—C2—C3—C4	0.0	C19—C14—C15—C16	0.0
C1—C2—C3—Cl1	−179.0 (2)	Br3—C14—C15—C16	173.9 (18)
C2—C3—C4—C5	0.0	C14—C15—C16—C17	0.0
Cl1—C3—C4—C5	179.0 (3)	C14—C15—C16—Cl4	−175 (2)
C2—C3—C4—Cl2	179.5 (3)	C15—C16—C17—C18	0.0
Cl1—C3—C4—Cl2	−1.5 (3)	Cl4—C16—C17—C18	175.1 (19)
C3—C4—C5—C6	0.0	C15—C16—C17—Cl3	−179.9 (11)
Cl2—C4—C5—C6	−179.5 (3)	Cl4—C16—C17—Cl3	−5(2)
C4—C5—C6—C1	0.0	C16—C17—C18—C19	0.0
C2—C1—C6—C5	0.0	Cl3—C17—C18—C19	179.9 (10)
Sn1—C1—C6—C5	179.6 (2)	C17—C18—C19—C14	0.0
C11—N1—C7—C8	0.0	C15—C14—C19—C18	0.0
N1—C7—C8—C9	0.0	Br3—C14—C19—C18	−173.8 (18)

Symmetry codes: (i) −*x*+1, *y*, −*z*+3/2.

### Hydrogen-bond geometry (Å, °)

**Table:**

*D*—H···*A*	*D*—H	H···*A*	*D*···*A*	*D*—H···*A*
N1—H1···Br1	0.88	2.58	3.315 (3)	142
